# Excessive alcohol consumption induces methane production in humans and rats

**DOI:** 10.1038/s41598-017-07637-3

**Published:** 2017-08-04

**Authors:** E. Tuboly, R. Molnár, T. Tőkés, R. N. Turányi, P. Hartmann, A. T. Mészáros, G. Strifler, I. Földesi, A. Siska, A. Szabó, Á. Mohácsi, G. Szabó, M. Boros

**Affiliations:** 1 Institute of Surgical Research, University of Szeged, Szeged, Hungary; 2 Department of Laboratory Medicine, University of Szeged, Szeged, Hungary; 3 MTA-SZTE Research Group on Photoacoustic Spectroscopy, University of Szeged, Szeged, Hungary; 4 Department of Optics and Quantum Electronics, University of Szeged, Szeged, Hungary

## Abstract

Various studies have established the possibility of non-bacterial methane (CH_4_) generation in oxido-reductive stress conditions in plants and animals. Increased ethanol input is leading to oxido-reductive imbalance in eukaryotes, thus our aim was to provide evidence for the possibility of ethanol-induced methanogenesis in non-CH_4_ producer humans, and to corroborate the *in vivo* relevance of this pathway in rodents. Healthy volunteers consumed 1.15 g/kg/day alcohol for 4 days and the amount of exhaled CH_4_ was recorded by high sensitivity photoacoustic spectroscopy. Additionally, Sprague-Dawley rats were allocated into control, 1.15 g/kg/day and 2.7 g/kg/day ethanol-consuming groups to detect the whole-body CH_4_ emissions and mitochondrial functions in liver and hippocampus samples with high-resolution respirometry. Mitochondria-targeted L-alpha-glycerylphosphorylcholine (GPC) can increase tolerance to liver injury, thus the effects of GPC supplementations were tested in further ethanol-fed groups. Alcohol consumption was accompanied by significant CH_4_ emissions in both human and rat series of experiments. 2.7 g/kg/day ethanol feeding reduced the oxidative phosphorylation capacity of rat liver mitochondria, while GPC significantly decreased the alcohol-induced CH_4_ formation and hepatic mitochondrial dysfunction as well. These data demonstrate a potential for ethanol to influence human methanogenesis, and suggest a biomarker role for exhaled CH_4_ in association with mitochondrial dysfunction.

## Introduction

Mammalian methanogenesis is regarded as a specific indicator of carbohydrate fermentation by the intestinal anaerobic microflora. It is also accepted that the bulk of methane (CH_4_) production is excreted via the lungs, and therefore changes in breath CH_4_ output are widely used for the diagnosis of certain gastrointestinal (GI) malabsorption conditions^1^. Nevertheless, the pulmonary route is not exclusive since a uniform CH_4_ release can be detected through the skin in healthy individuals^2^. It is also noteworthy that two distinct human populations are revealed with the diagnostic breath tests, CH_4_-producers and non-producers, when production is usually being defined as a >1 ppm increase above the atmospheric CH_4_ concentration^3^. Besides, a recent study using stable carbon isotope signatures provided clear evidence that the exhaled CH_4_ levels are always above the inhaled CH_4_ concentration, supporting the idea that all individuals might produce endogenous CH_4_ which cannot be detected by conventional analytical techniques^4^.

Of interest, the *in vivo* methane formation cannot be restricted to prokaryotes because various *in vitro* and *in vivo* experimental data have established the possibility of biotic, non-bacterial generation of CH_4_ under various stress conditions in plants and animals also^5–10^. In this line, significant *in vivo* CH_4_ release was demonstrated in a rodent model of chemical asphyxiation, after chronic inhibition of the activity of mitochondrial cytochrome c oxidase^11^.

Collectively these findings suggested us that CH_4_ excretion in mammals may reflect bacterial and non-bacterial methanogenesis as well. In this context, the primary objective of the present study was to provide evidence for the opportunity of alternative, non-conventional CH_4_ production in humans. Since an increased ethanol input is a common way to induce hepatic oxido-reductive imbalance in man^12^, we set out to investigate the possibility of CH_4_ generation in previously non-methane producer volunteers consuming high doses of ethanol. For the detection of *in vivo* CH_4_ output we employed a high sensitivity, near-infrared laser technique-based photoacoustic spectroscopy (PS) system, which has previously been validated for real-time measurements of CH_4_ emissions in human and animal studies^11, 13^.

A further aim was to extend the scope of the human protocol in a comparable animal model of ethanol challenge. It has been shown that excessive ethanol intake may lead to a transient failure of the mitochondrial electron transport chain (METC) leading to oxidative membrane damage^14–17^ in humans and rodents^18^, thus we set out to collect analogous animal data on ethanol-induced CH_4_ generation in association with mitochondrial functional failure in the liver and hippocampus tissue.

The functional consequence of endogenous CH_4_ production is subject of debate. We hypothesized that if CH_4_ production is induced from target cellular components, a greater understanding of a process that modulates this response would be of interest. L-alpha-glycerylphosphorylcholine (GPC) is a water-soluble deacylated metabolite of membrane-forming phosphatidylcholine (PC) and a source of choline^19–21^. Interestingly, significantly lower concentrations of hepatic GPC have been reported after experimental haemorrhagic shock, a prototype of systemic hypoxia and mitochondrial dysfunction^22^ and our earlier *in vivo* findings demonstrated that GPC is protective against several signs of hypoxia- or redox-imbalance-induced tissue injuries^11, 23, 24^. Thus, in the next part of the rat study we examined the hypothesis that GPC may influence CH_4_ production through the modulation of alcohol-induced mitochondrial dysfunction.

## Results

### Human breath CH4 analysis

**Figure 1:**
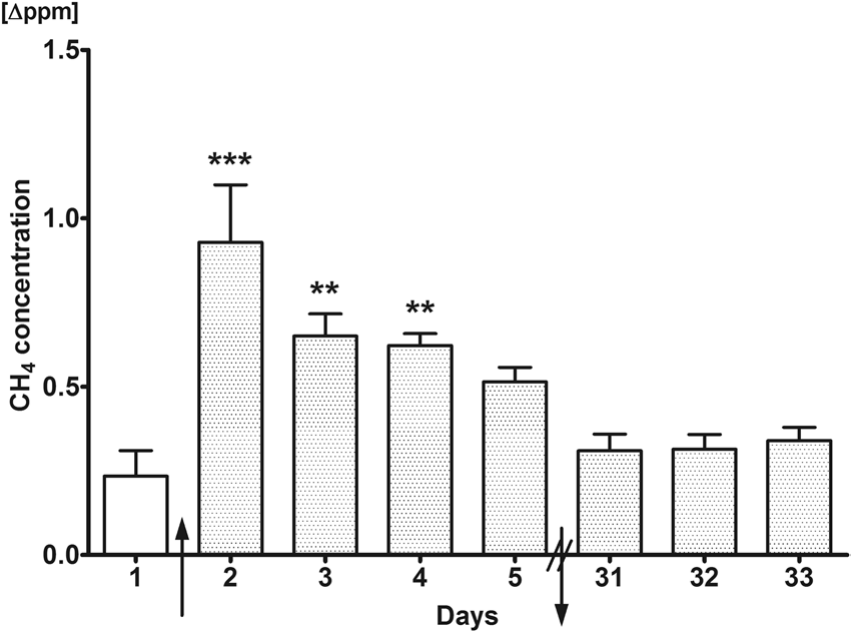
Exhaled CH_4_ production profile of humans during the course of the study. Exhaled CH_4_ production on days 1–5 and 31–33 of the investigation is depicted. During the first measurement on day 1, all volunteers were untreated. Alcohol consumption induced significant CH_4_ production by the 2^nd^ day compared to the 1^st^ day, and this level decreased remarkably by the 5^th^ day. Mean values and standard error of mean (SEM) are given; **p < 0.01 and ***p < 0.001 *vs*. day 1. The arrows pointing upwards and downwards represent start and end of alcohol intake, respectively. Statistics: one-way ANOVA with Dunnett’s multiple comparison test.

The possibility of ethanol-induced methanogenesis was monitored in previously non-CH_4_ producer individuals under standardized circumstances. The breath CH_4_ concentration was significantly elevated by approx. 0.9 Δppm on day 2 (0.93 ± 0.17 Δppm), day 3 (0.65 ± 0.07 Δppm) and day 4 (0.62 ± 0.04 Δppm), as compared to the baseline (day 1, 0.23 ± 0.08 Δppm). The CH_4_ output was decreasing by day 5 (0.51 ± 0.04 Δppm) but remained significantly higher over the baseline. When the breath CH_4_ analysis was repeated 31 days later, statistically significant CH_4_ production was not detected *(see* Fig. 1 *)*.

### Liver-specific enzymes

**Figure 2:**
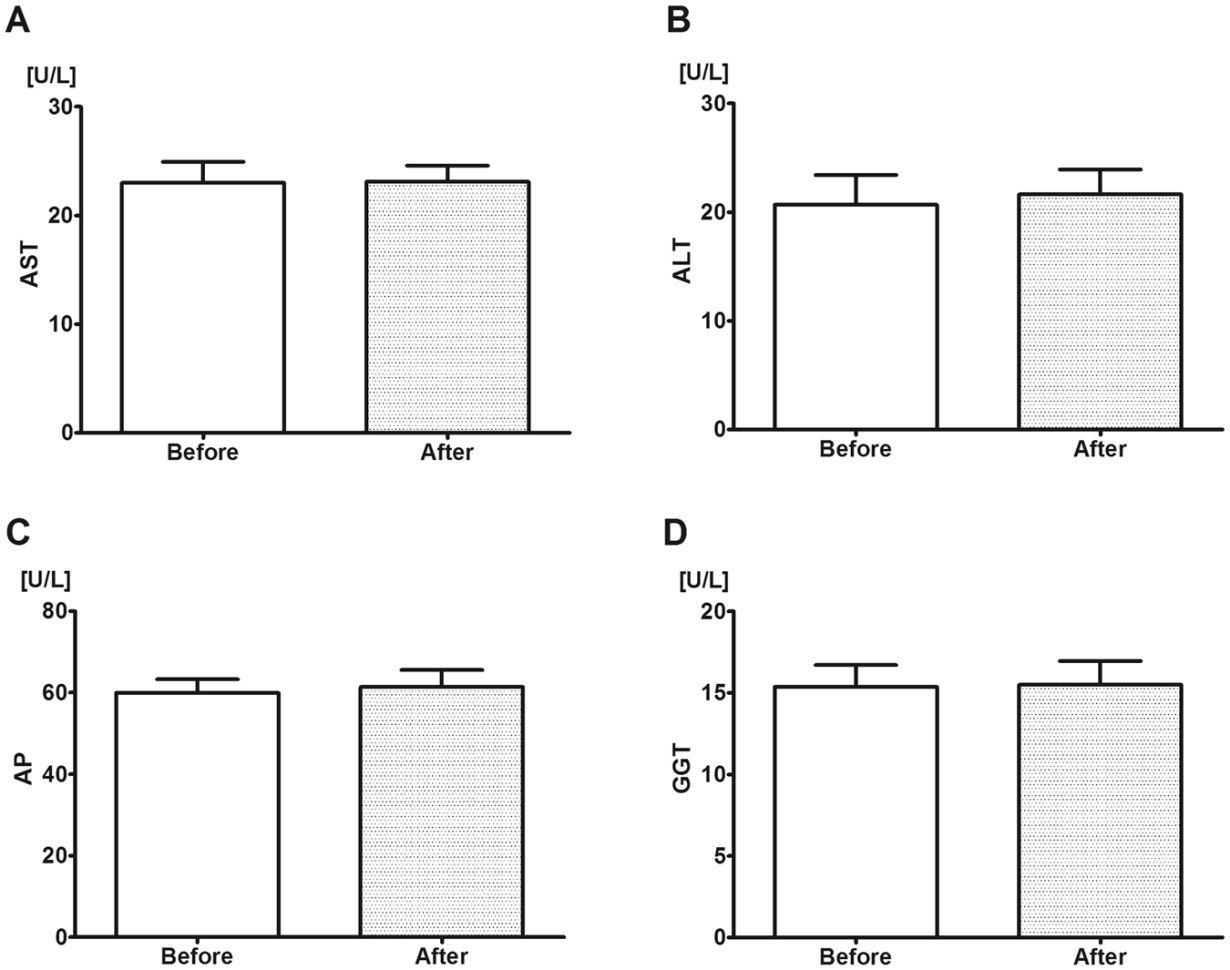
Liver-specific enzyme levels before and after alcohol consumption in humans. Enzyme levels were determined in plasma from blood samples taken on days 1 (control, left boxes) and 5 (last day of alcohol consumption, right boxes) of the investigation. During the first measurement, all volunteers were untreated. **(A)** Aspartate aminotransferase (AST), **(B)** Alanine aminotransferase (ALT), **(C)** Alkaline phosphatase (AP) and **(D)** Gamma glutamyl transferase (GGT) levels. Alcohol consumption did not alter enzyme levels. Mean values and standard error of mean (SEM) are given; p < 0.05 *vs*. day 1 (control). Statistics: Paired-test.

Clinically established enzyme markers were used to monitor the liver function before and after the 4-days alcohol intake. Statistically significant changes were not observed in these parameters (see Fig. 2 *)*.

### CH_4_ release in rats

**Figure 3:**
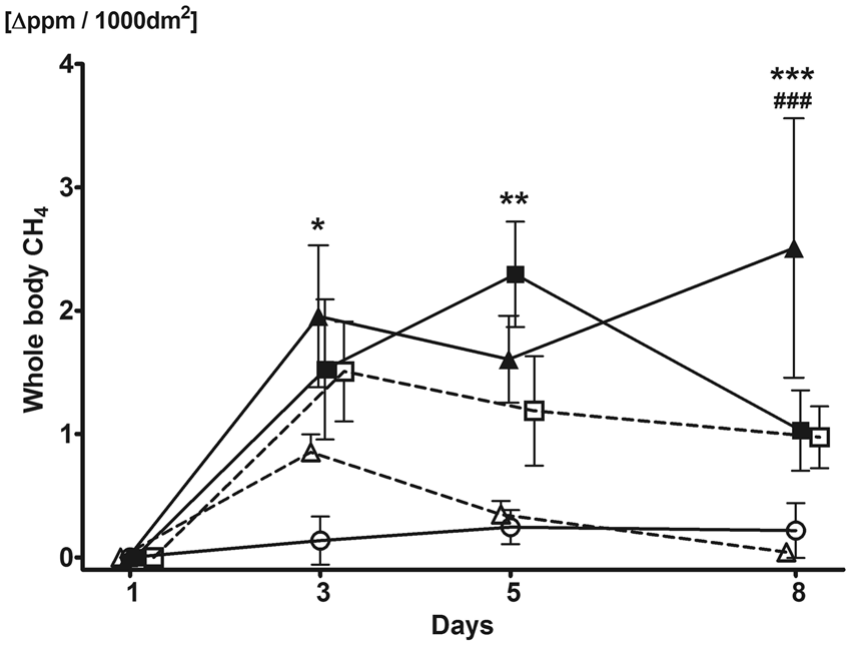
Whole-body CH_4_ release of rats. Whole body CH_4_ production was measured on days 1, 3, 5, and 8 of the investigation. Ethanol feeding induced significant CH_4_ production by the 3^rd^ day in the 1.15 g/kg/day alcohol-treated group and by the 5^th^ day in the 2.7 g/kg/day alcohol-treated group, compared to control group. However, in the GPC-treated groups no increase was observed as compared to controls. Empty circles with continuous line relates to control group, black triangles with continuous line to the 1.15 g/kg/day alcohol-treated group, empty triangles with dashed line to the 1.15 g/kg/day alcohol + GPC-treated group, black squares with continuous line to 2.7 g/kg/day alcohol-treated group, and empty squares with dashed line to the 2.7 g/kg/day alcohol + GPC-treated group. During the measurement on day 1, all the rats were untreated. Mean values and standard error of mean (SEM) are given; *p < 0.05, **p < 0.01 and ***p < 0.001 *vs*. Control group, ^**###**^p < 0.001 *vs*. corresponding GPC-treated groups. Statistics: Two-way ANOVA, Bonferroni post-hoc test.

In a forward step, we followed the whole-body CH_4_ profile of rats during several days of alcohol consumption (see Fig. 3 *)*. This study has shown increased CH_4_ output (1.85 ± 0.48 Δppm/1000 dm^2^) compared to the control group on 3 day after an intake of 1.15 g/kg/day ethanol and this level was sustained until the end of the 8-days observation period (2.47 ± 1.29 Δppm/1000 dm^2^). However, no elevation in CH_4_ levels was seen in the 1.15 g/kg/day ethanol + GPC treated group compared to controls. By day 8, there was already a significant difference between the group with 1.15 g/kg/day ethanol without GPC (2.47 ± 1.29 Δppm/1000 dm^2^) and the group with GPC treatment (0.04 ± 0.04 Δppm/1000 dm^2^).

By day 5, the 2.7 g/kg/day-ethanol load increased the whole-body generation of CH_4_ (2.3 ± 0.43 Δppm/1000 dm^2^), and the high CH_4_ production was kept up until the end of the 8-days experiments (1.03 ± 0.33 Δppm/1000 dm^2^). Similar to the low-dose ethanol + GPC, CH_4_ release in the 2.7 g/kg/day ethanol + GPC-treated group did not differ from controls. No changes were observed in the untreated control group during the observation period (day 8: 0.23 ± 0.18 Δppm/1000 dm^2^) as compared to baseline levels on day 1.

### Mitochondrial function in the rat liver and hippocampus

**Figure 4:**
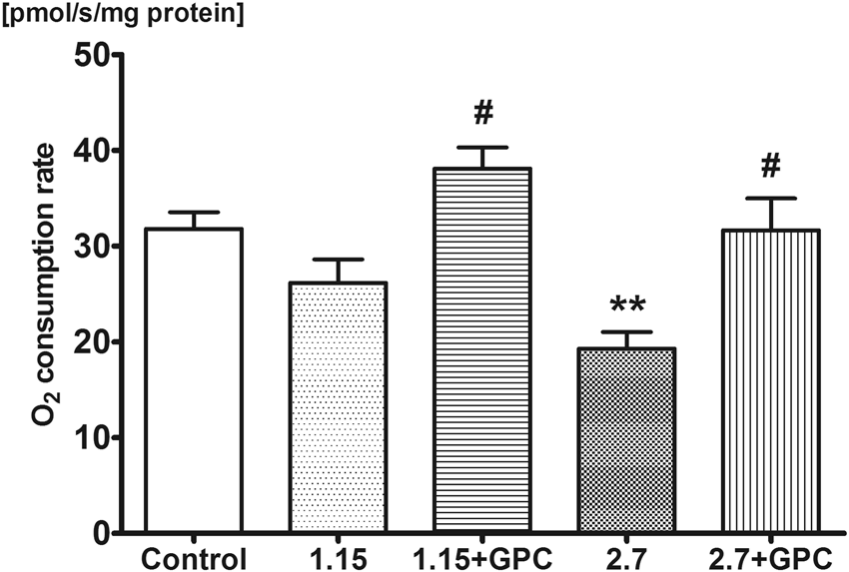
Oxygen consumption of rat liver mitochondria. O_2_ consumption rate of mitochondria was assessed in liver homogenate after addition of 0.5 μM rotenone, 10 mM succinate and 2.5 mM ADP on day 9 of investigation. The 2.7 g/kg/day ethanol-fed group had decreased mitochondrial function compared to control, whereas the GPC treatment prevented the decline of mitochondrial oxygen consumption, demonstrated by higher levels compared to both non-GPC treated ethanol-fed groups. Mean values and standard error of mean (SEM) are given; **p < 0.01 *vs*. Control, ^#^p < 0.05 *vs*. corresponding GPC-treated groups. Statistics: One-way ANOVA with Bonferroni post-hoc test.

**Figure 5:**
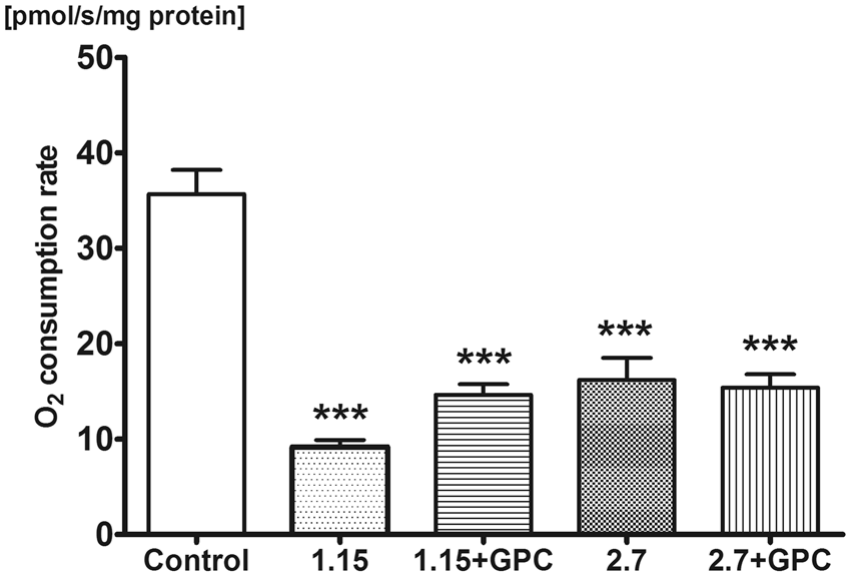
Oxygen consumption of rat hippocampus mitochondria. O_2_ consumption rate of mitochondria was assessed in hippocampus homogenate after addition of 0.5 μM rotenone, 10 mM succinate and 2.5 mM ADP on day 9 of investigation. Mitochondrial respiratory capacity deteriorated upon alcohol intake regardless of GPC treatment. Mean values and standard error of mean (SEM) are given; ***p < 0.001 *vs*. Control. Statistics: One-way ANOVA with Bonferroni post-hoc test.

The oxidative phosphorlylation capacity of METC in liver and brain homogenates was analyzed with high-resolution respirometry with saturating succinate and ADP concentrations (see Figs 4 and 5).

In the liver, intake of 1.15 g/kg/day alcohol did not influence mitochondrial oxygen consumption as compared to control group (26.17 ± 2.45 pmol/s/mg protein *vs* 31.79 ± 1.74 pmol/s/mg protein), while 2.7 g/kg/day ethanol challenge resulted in a significant reduction in oxidative phosphorylation capacity (19.29 ± 1.73 pmol/s/mg). Dietary supplementation of GPC lead to an increase in respiration in both groups compared to their non-GPC-treated counterparts (1.15 g/kg/day alcohol + GPC: 38.08 ± 4.99 pmol/s/mg protein; 2.7 g/kg/day alcohol + GPC: 31.66 ± 8.11 pmol/s/mg protein).

The alcohol challenge significantly compromised the mitochondrial oxygen uptake in the hippocampus as well (Figs 4 and 5), and GPC treatment did not influence the change in this tissue.

### Liver-specific enzyme levels in the rat plasma

**Figure 6:**
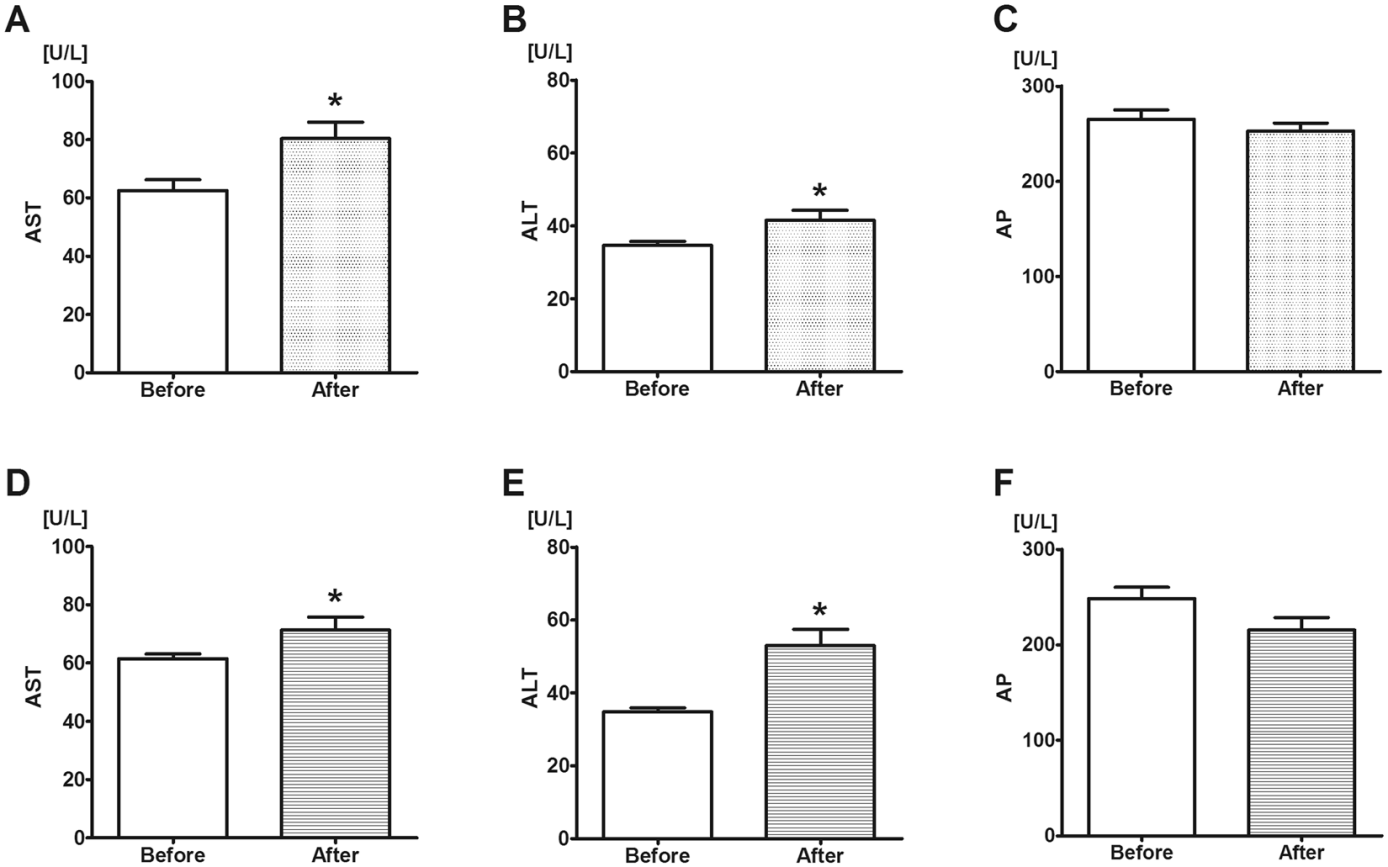
Liver-specific enzyme levels before and after alcohol consumption in rats. Enzyme levels were determined in plasma from blood samples taken on days 1 (untreated control, left boxes) and 8 (last day of alcohol treatment, right boxes) of the investigation. The 1.15 g/kg/day alcohol (panels A–C) and 1.15 g/kg/day alcohol + GPC-fed group (panels D–F) were monitored with same method. Both Aspartate aminotransferase (AST, panels A and D) and Alanine aminotransferase (ALT, panels B and E) levels increased significantly as compared to baseline. Alkaline phosphatase (AP, panels C and F) levels did not change. Mean values and standard error of mean (SEM) are given; *p < 0.05 *vs*. day 1 (Control). Statistics: Paired t-test.

The plasma levels of liver-specific enzymes were monitored before and after the 1.15 g/kg/day oral alcohol intake regime to obtain comparable data with the human study. The ALT and AST plasma levels increased from 34.6 U/L to 41.5 U/L and from 62.5 U/L to 80.3 U/L, respectively, while the AP levels did not change. The plasma enzymes levels in the GPC-treated group showed similar changes over time, as the values of ALT rose from 34.8 U/L to 53 U/L and the concentration of AST elevated from 61.5 U/L to 71.3 U/L, while the AP levels did not change (see Fig. 6). GGT was not measured in rat plasma samples due of methodological limitations.

## Discussion

Methanogenesis in humans has been considered an exclusive attribute of methanogenic Archaea, a group well distinguished from the usual bacteria and the eukaryotes. These strictly anaerobic inhabitants of the GI tract are producing CH_4_ from decomposing organic matter through hydrogenotrophic, methylotrophic and acetotrophic classes of methanogenesis^25^. Nevertheless, to-date a number of studies have demonstrated the generation of non-bacterial CH_4_ also in aerobic living systems^5, 6, 9^, and it has also been suggested that the CH_4_-producing phenomenon can be in association with transient losses of redox homeostasis^7, 8^. Indeed, the administration of sodium azide (NaN_3_) leads to an increasing emanation of endogenous CH_4_ in plant and animal cells and whole animal organisms^11, 26^. The main effect of NaN_3_ in eukaryotes is its direct and irreversible binding to the heme cofactor of cytochrome c oxidase, the final electron acceptor of the METC; thus it can also be considered a tool via which to study mitochondrial oxido-reductive stress.

This study reports a combination of observational and experimental data on ethanol-induced CH_4_ generation, and to our knowledge, these results provide the first evidence for increased *in vivo* CH_4_ output after such manipulation. It should be added that analysis of exhaled breath samples is widely used in the clinical practice for the screening of patients, where the detection of CH_4_ concentration changes relative to other gaseous compounds can promote the diagnosis; the amount of exhaled H_2_ and CH_4_ proves that a carbohydrate substrate has been exposed to bacterial fermentation^1, 27^. However, Keppler *et al*. demonstrated that CH_4_ was produced by all of the human subjects studied^4^. Using laser absorption spectroscopy and stable carbon isotopes to precisely measure exhaled and inhaled CH_4_ concentrations in various age groups, evidence was presented that humans might also produce CH_4_ endogenously in cells^4^. Our results do not exclude the role of microorganisms or do not negate the validity of breath-testing-based diagnostic methods but also suggest that other factors and pathways need to be also taken into account in the background of mammalian CH_4_ generation. One of the possibilities is alcohol consumption.

In our studies the relatively high ethanol intake was followed by significant whole-body CH_4_ release in previously non-producer animals, and also in humans, the non-CH_4_ producer phenotype was “transformed” to CH_4_ producer. These parallel results do suggest that CH_4_ production may occur *in vivo* independently from the gut microbiome, but the bacterial origin cannot be excluded with certainty (see Supplementary Figure 1, as well). In the complex ecosystem of the GI tract, methanogens are compelled to compete with other microorganisms, such as sulphate-reducing bacteria for the common substrates in the human colon^28^. Bacterial enzymatic CH_4_ is formed when CO_2_ is reduced by electron donor H_2_ produced by anaerobic fermentation. Ethanol can be substrate for some methanogens as demonstrated in a recent *in vitro* study with different peatland methanogenic bacteria to use ethanol and acetate as substrate^29^. Although some studies examined the relation between alcohol consumption and gut microbiome composition in humans, specific information on the response of methanogenic bacteria is lacking^30, 31^. Nevertheless, prolonged alcohol consumption can alter the composition and function of colon microbiome, and in turn, the gut microbiome can have a profound impact on alcohol metabolism as well^30^.

In the clinical laboratory practice breath CH_4_ levels are usually analyzed by means of gas chromatography (GC) and GC-mass spectrometry, but these traditional methods have technical limitations^32^. The PAS system was tested for whole-body and single breath CH_4_ analyses in previous surveys^11, 13^, and the online detected CH_4_ changes have proved to be reproducible and highly specific for CH_4_ in a wide dynamic range^13^.

Today it is accepted that the CH_4_ concentration in the breath is usually greater than 1 ppm in 30–60% of humans^3, 33^. Our data suggest that large differences in breath gas analysis data are presumably not only due to the variations in the personal background, bacterial strains, sampling and analysis techniques, but other cause as well. Nevertheless, if nonbacterial CH_4_ is mixed with bacterial CH_4_ production, this addition is not easy to identify and recognize. To elucidate whether a similar process of CH_4_ generation exist in humans and rats, we have chosen a standardized, reversible mitochondrial stress induction procedure. Indeed, hepatic and hippocampal METC functions were affected significantly as evidenced by the direct mitochondrial analysis in the rat. It is known that alcohol consumption influences the activities of respiratory chain complexes II and III in the mouse brain^34^, and decreases the state III respiration and the respiratory control ratio in the liver of ethanol-fed animals^35^. Here we demonstrate that the complex II-linked state III respiration decreased significantly both in the liver and the hippocampus as compared to the control group. The ethanol challenge decreased the complex II-linked oxygen consumption of liver mitochondria in a dose-dependent manner, while this inhibitory effect was much more pronounced in the hippocampus already after the lower ethanol dosage. What is more, GPC administration did not improve the respiratory capacity of hippocampal mitochondria. GPC is a mitochondria-targeted compound which enhances mitochondrial oxygen consumption in hepatocytes^36^ and it has been shown that GPC can rapidly cross the blood-brain barrier^20, 37^. A possible explanation for these differences is provided by the higher susceptibility of hippocampal neurons and/or mitochondria towards the ethanol input and the organ-specific impact of ethanol-induced oxidative stress on mitochondria of these cell types^38, 39^.

The plasma concentrations of liver-specific enzymes were measured to provide an overview of the metabolic function of the liver after the transient ethanol challenge. AST and ALT are located normally in the cytoplasm of hepatocytes, and upon membrane damage a portion of the intracellular aminotransferases leak into the extracellular fluid^40^. The plasma levels of AST and ALT did not change in human volunteers after the 4 days of regular alcohol consumption, while a slight but significant (approx. 10–30%) increase in AST and ALT levels was detected in ethanol-fed rats. Inter-species variations and liver-specific reactions in ethanol metabolism and tolerance cannot be excluded, but these data suggest that the severity of alcohol-induced metabolic stress only slightly exceeded the hepatocellular injury threshold, and the model was associated only with a reversible hepatic functional imbalance in both species.

A transient, significant CH_4_ production was the appropriate answer to ethanol intake in both experimental series, and there is supporting data in the literature that METC dysfunction can be accompanied by CH_4_ generation^8^. The initial *in vitro* studies led to the proposal that electrophilic methyl groups (EMG) bound to positively charged nitrogen moieties (as in choline molecules) may potentially act as electron acceptors, during reductive stress conditions and that these reactions may entail the generation of CH_4_^5, 6^. Liver NAD^+^/NADH + H^+^ changes are characteristically increased after alcohol intake^41^. Most of the alcohol is metabolized to acetaldehyde by alcohol dehydrogenase in the cytoplasm of hepatocytes and the catalyzer is NAD^+^ which is transformed to NADH + H^+^. Acetaldehyde is transformed further to carboxylic acid by aldehyde dehydrogenase meanwhile the system uses more NAD^+^ and water. The resultant increasing NADH + H^+^ level leads to reductive stress^41, 42^. In addition, alcohol exposure can both affect gene expressions and activity of S-adenosylhomocysteine hydrolase^42, 43^, and may change the methionine metabolism leading to intracellular S-adenosylmethionine (SAM) and S-adenoslyhomocystenine (SAH) increases^42^. Thus, the ethanol-induced imbalance of NADH can be an important key to the genesis of SAH and SAM. Moreover, the system produces CH_4_ during this reaction.

It should be added that reversible methionine oxidation could be a mechanism of redox- regulation, which involves the action of methionine sulfoxide reductases (MSR) whose main function is to protect membranes from oxidative damage^44^. Nevertheless, methionine and methionine sulfoxide exhibit high CH_4_-formation ability *in vitro*, without enzymes to catalyze the reaction^44^, and the sulfur-bonded CH_3_ group in methionine was unambiguously identified by stable isotope labeling techniques as the carbon precursor of the CH_4_ molecule in fungi under aerobic conditions^45^. In this line, a chemical reaction was recently described by Althoff *et al*. where CH_4_ is readily formed from the S-CH_3_ groups of organosulphur compounds in a model system containing iron(II/III), H_2_O_2_ and ascorbate that uses organic compounds with heterobonded CH_3_ groups for the generation under ambient (1.000 mbar and 22 °C) and aerobic (21% O_2_) conditions^46, 47^.

The role of choline in aerobic CH_4_ formation was also substantiated in previous *in vivo* and *in vitro* experiments. Exogenous phosphatidylcholine (PC) and choline metabolites can suppress the methanogenic reaction^8^, and similarly, when GPC, a water-soluble, deacylated PC derivative was administered in NaN_3_-induced chemical hypoxia, the extent of CH_4_ generation was reduced^11^. GPC is a bioavailable source of choline^48^, which can be directly or indirectly involved in the preservation of membranes in ischemic tissue damage^49^. The exact mechanism of GPC interference with the liver oxidative phosphorylation capacity is still unclear. Nevertheless, choline metabolites are involved in the preservation of the structural integrity of cellular membranes^50^. More importantly it has been shown that a choline deficiency impairs complex I (NADH dehydrogenase)-linked respiration^51^. Indeed, in our study exogenous GPC protected against the ethanol-induced METC dysfunction in the rat liver, the primary target of alcohol-induced oxido-reductive stress. There is evidence suggesting a direct action of GPC on mitochondrial complex II-linked respiration. After oral intake GPC is hydrolysed by phosphodiesterases in the intestinal mucosa^20^ and in liver as well^52^ producing choline and glycerol-3-phosphate (G3P). G3P can be directly oxidised by G3P-shuttle (G3P dehydrogenase) feeding FADH2 to mitochondrial complex II and enhancing mitochondrial oxidative phosphorylation. We did not test to see whether GPC supplementation improves complex I-linked respiration, but this aspect definitely deserves elucidation in the future. Since it has been shown that GPC rapidly delivers both choline and glycerol to the brain across the blood–brain barrier^20, 51^ we have expected similar effectiveness in the central nervous system too, but this approach did not improve the respiratory capacity of hippocampal mitochondria. We speculate that maybe even the lower dose of ethanol did damage the sensitive pyramidal cells of the hippocampus so severe that GPC could not prevent loss mitochondrial respiratory function. As the reason for this finding is unclear, further studies with different doses and timing are clearly needed in this respect^23^.

Before making the final conclusions, it is important to note the limitations of the experimental setup. Firstly, the exact pathway through which CH_4_ is liberated after the *in vivo* alcohol load remained basically unexplored and several sources, bacterial and non-bacterial, can be postulated. In this sense it is still unknown whether the findings in this experimental setup are applicable to other conditions, and the therapeutic significance of GPC administration should be reinforced and reproduced in further studies. Nevertheless, we have provided evidence that transient, high ethanol input leads to significant increases in CH_4_ formation in previously non-CH_4_ producer humans, and a similarly high CH_4_ formation occurs in ethanol-consuming rats, in association with a mitochondrial dysfunction in the liver. Biomarkers are essential clinical tools through which to gain an understanding of human biology or the responses to therapies, and CH_4_ is regarded as disease-specific routine biomarker. Therefore this type of alternative CH_4_ formation might be an interesting focus for diagnostic and therapeutic strategies in IR episodes. Much remains unknown about hypoxic reactions and the fate of intracellular CH_4_ is an open question, but the results presented indicate a biomarker role for CH_4_.

## Materials and Methods

### Human protocol

All procedures in this study involving human subjects were in accordance with the Declaration of Helsinki and approved by the Human Investigation Review Board of the University of Szeged, Albert Szent-Györgyi Clinical Centre (Institutional Ethical Board approval No. 49/B-165/2014). The participants signed an informed consent and all approved the publication of the data obtained. Healthy men (11 volunteers) with no known underlying disease, 25 to 30 years age (average weight 82 ± 13.1 kg) were included in the study. The participants underwent a general health check with repeated breath analyses (see later). The exclusion criteria were CH_4_ producer status, smoking, regular alcohol intake or ethanol dependency, competitive sports, liver disease and diabetes. Alcohol intake was not allowed for 3 days prior to the study and the enrolled subjects underwent a baseline measurement of breath ethanol.

On day 1 of the protocol control breath CH_4_ measurements were carried out, and then the subjects consumed 1.15 g/kg/day alcoholic beverage (200 ml whisky, 40 vol% ethanol content) which was repeated between 6 pm and 7 pm on 4 consecutive days. Breath CH_4_ measurements were performed every day at a preset time (between 8 am and 9 am) with empty stomach, before the first meal. In order to assess the activity of liver-specific enzymes, 3 ml peripheral venous blood samples were taken into EDTA-containing tubes before the start of alcohol consumption protocol and after the end of the last breath CH_4_ measurement. The breath CH_4_ analysis was repeated 31 days later for 3 consecutive days when the subjects did not consume alcohol for at least 3 days before the measurements.

### CH_4_ analysis setup

The CH_4_ concentration of breath samples was detected by PAS as described previously^13^. PAS is a special mode of spectroscopy which measures optical absorption indirectly via the conversion of absorbed light energy into acoustic waves. The amplitude of the generated sound is directly proportional to the concentration of the absorbing gas component. The light source of the system is a near-infrared diode laser that emits around the CH_4_ absorption line at 1650.9 nm with an output power of 15 mW (NTT Electronics, Tokyo, Japan). Cross-sensitivity for common components of breath and ambient air were repeatedly examined, and no measurable instrument response was found for several vol % of CO_2_ or H_2_O vapour. The narrow line width of the diode laser provides high selectivity; the absorbance of CH_4_ is several orders of magnitude greater than that of H_2_O, CO_2_ or CO at 1.65 μm, the wavelength we used. The device was previously calibrated with various gas mixtures prepared by dilution of 100 ppm CH_4_ in synthetic air (Messer, Budapest, Hungary), and it has a dynamic range of 4 orders of magnitude; the minimum detectable concentration of the sensor was found to be 0.25 ppm (3σ), with an integration time of 12 s^13^.

### Breath sampling in humans

The subjects were asked to breathe normally and the expired air was directed into a 200 cm^3^ glass flask. The optimal time for the attainment of a steady CH_4_ concentration proved to be between 1 and 3 min with a flow rate of 30 cm^3^/min. The room air CH_4_ level was determined and used as baseline in the calculations of *in vivo* CH_4_ emission. CH_4_ production is given in Δppm, referring to the net exhaled CH_4_ concentration, where the CH_4_ concentration of the ambient air was deducted from exhaled CH_4_ concentration.

### Rat study

The experiments were performed on male Sprague-Dawley rats (190–300 g bw) in accordance with the National Institutes of Health guidelines on the handling and care of experimental animals and EU Directive 2010/63 for the protection of animals used for scientific purposes. The study protocol was reviewed by the National Scientific Ethical Committee on Animal Experimentation (National Competent Authority of Hungary) and was approved by the Animal Welfare Committee of the University of Szeged (V/148/2013).

Prior to the start of the experiments control whole-body CH_4_ measurements were carried out (see details later) and non-producer CH_4_ status (less than 0.3 ppm) animals were included into the study.

A pilot dose-response study was performed with 6 non-CH_4_ producer rats. These animals were fed *per os* with alcoholic solutions (40% whisky in gavage up to 2.86 ml/kg/day) for 8 days. The dosage was equivalent to 1.15 g/kg/day ethanol intake which was employed in the human study.

After receiving standard dose-responses, 24 non-CH_4_ producer rats were randomly allocated into four groups. Group 1 (n = 6) served as non-treated control, in Group 2 (n = 6) the animals received 1.15 g/kg/day alcohol with supplemented oral GPC feeding (0.8% GPC-enriched diet, Ssniff Spezialdiäten GmbH, Soest, Germany), in Group 3 (n = 6), the animals received 7.5 ml/kg/day 40% alcoholic solution *per os* for 8 days in daily gavage. This dose is equivalent to 2.7 g/kg/day ethanol intake, which has been reported to lead hepatic oxido-reductive stress reproducibly in rats^47–49^. In Group 4 (n = 6), the identical alcohol input (7.5 ml/kg/day) was supplemented with oral GPC feeding for 8 days.

The whole-body CH_4_ emission of the animals was measured in a specially-designed closed sampling chamber with an internal volume of 2510 cm^3^ as reported previously^13^. Prior to the experiments the CH_4_ concentration in the chamber was determined and used as baseline value. By means of a membrane pump (*Rietschle Thomas*, Puchheim, *Germany*) and a mass-flow controller, a sample of the gas from the chamber was drawn through the photoacoustic cell of the measuring device via a stainless steel tube. The rat was next placed into the chamber, which was then sealed. It was found that a period of 10 min was sufficient to reach the level of CH_4_ released by the animal to be measured reliably and reproducibly in an extraction sample. Accordingly, a sample of the chamber gas was analysed exactly 10 min after the animal had been placed in the chamber. The rat was then removed and the measurements were repeated on the subsequent day. The chamber was thoroughly flushed with room air before the next rat was placed in. The whole-body CH_4_ emission of the animals was calculated as the difference in the baseline CH_4_ concentration and the 10 min sample and referred to the body surface (F (dm^2^) = 10*S^0.75^ kg).

### Analysis of mitochondrial function in the rat

After the last gas measurements the animals were anesthetized with 5% chloral hydrate (375 mg/kg), and approx. 50 mg tissue biopsies were subsequently taken to determine the mitochondrial function in the liver and the hippocampus. The tissue samples were immediately placed into 2 ml of PBS-based homogenization buffer containing 0.25 M saccharose, 10 mM Tris, 0.5 mM EDTA and 0.05% BSA. For determination of the respiratory activity of mitochondria high-resolution respirometry (Oxygraph-2k, Oroboros Instruments, Innsbruck, Austria) was used. 50 μl tissue homogenates were incubated with the same buffer in the measuring chamber for 30 sec to record state I respiration. At the end of state I, the steady-state basal oxygen consumption (respiratory flux) was detected. The complex II-linked respiration rate (state II) was determined after addition of complex I inhibitor 0.5 μM rotenone, and after reaching the plateau of flux, and additional administration of 10 mM succinate. After reaching the plateau of flux, 2.5 mM ADP was added for determination of oxidative phosphorlyation capacity of the samples (state III respiration). The oxygen consumption rates were referred to the protein content of the sample.

### Liver-specific enzymes

Serum liver-specific enzymes were measured by commercially available diagnostic kits (Roche, Mannheim, Germany). Aspartate aminotransferase (AST) was determined by UV kinetic method using alpha-ketoglutarate and L-aspartate as substrates. Alanine aminotransferase (ALT) was measured by UV kinetic method using alpha-ketoglutarate and L-alanine as substrates. Gamma glutamyl transferase (GGT) was measured by enzymatic colorimetric method using L-gamma-glutamyl-3-carboxy-4-nitroanilide and glycylglycine substrates. Alkaline phosphatase (AP) activity was measured 4-nitrophenyl phosphate as substrate and 2-amino-2-methyl-l-propanol as buffer on a Modular P800 (Roche) chemistry analyzer. IFCC standardized UV kinetic method without pyridoxal phosphate activation were used for AST and ALT, IFCC standardized colorimetric assay was used for the AP measurements.

### Statistical analysis

Data analysis was performed with the statistical software package GraphPad Prism 5.01 for Windows (GraphPad Software, La Jolla, California, USA). For time-dependent differences within groups repeated measures one-way ANOVA with Dunnett’s multiple comparison test and for time-dependent differences of multiple groups two-way ANOVA, followed by Bonferroni post-hoc test was applied. Two-tailed paired t-test was used to compare values before and after treatments within groups. One-way ANOVA with Bonferroni post-hoc test was applied to compare multiple groups. In the figures, mean values and standard error of mean (SEM) are given, p values < 0.05 were considered significant.

## Electronic supplementary material

Supplementary Dataset 1
