# Supplementary material for: Excessive alcohol consumption induces methane production in humans and rats
Source: Sci Rep. 2017 Aug 4;7:7329. doi: 10.1038/s41598-017-07637-3 (PMC5544731; doi:10.1038/s41598-017-07637-3)
Supplement: Supplementary file 1 — Supplementary Dataset 1 [file 41598_2017_7637_MOESM1_ESM.doc]

**Excessive alcohol consumption induces methane production in humans and rats**

**E. Tuboly^1+^, R. Molnár^1+^, T. Tőkés^1^, R. N. Turányi^1^, P. Hartmann^1^, A. T. Mészáros^1^, G. Strifler^1^, I. Földesi^2^, A. Siska^2^, A. Szabó^3,4^, Á. Mohácsi^3,4^, G. Szabó^3,4^, M. Boros^1*^**

*^1^ Institute of Surgical Research, University of Szeged, Hungary*

*^2^* *Department of Laboratory Medicine, University of Szeged, Hungary*

*^3^MTA-SZTE Research Group on Photoacoustic Spectroscopy, University of Szeged, Hungary*

*^4^Department of Optics and Quantum Electronics, University of Szeged, Hungary*

**^+^***These authors contributed equally to this paper*

***Corresponding author:**

Mihály Boros MD, PhD, DSc

Institute of Surgical Research, University of Szeged**,** Szőkefalvi-N B u 6 H-6720 Szeged, Hungary

**E-mail**: boros.mihaly@med.u-szeged.hu

**Phone:** +36-62-545103

**Fax:** +36-62-455743

**Running head**: Ethanol-induced methane generation

**Supplementary Information**

A supplementary rat study was designed and performed in accordance with the National Institutes of Health guidelines on the handling and care of experimental animals and EU directive 2010/63 for the protection of animals used for scientific purposes. The study protocol was reviewed by the National Scientific Ethical Committee on Animal Experimentation (National Competent Authority of Hungary) and was approved by the Animal Welfare Committee of the University of Szeged (V/148/2013).

In this additional series alcohol-fed animals (2.7 g/kg/day *per os* for 8 days, n=6) were treated with antibiotics (rifaximin 10 mg kg^-1^ day^-1^, *per os*, Alfa Wasserman, West Caldwell, NJ, USA) for 11 days, the first dose being administered 3 days before the start of the oral alcohol treatment. This procedure resembles the human clinical practice of targeting the gastrointestinal bacterial flora. The rifaximin dose was chosen with regard to the minimal inhibitory concentration (25 µg ml^-1^) at which 50% of the strains are inhibited (Ref. Finegold SM, *Antimicrob Agents Chemother,* 53:281-6; 2009), the applied dosage (i.e. 2.5 mg ml^-1^) was approx. 100-times higher than the overall minimal inhibitory concentration.

We had to include control groups in adherence to the 3Rs in animal research, thus the new experimental data were analysed and compared to the control data obtained in the original rat study (n=6) with 2.7 g/kg/day ethanol feeding and n= 6, untreated control). The results are summarized in the figure below.


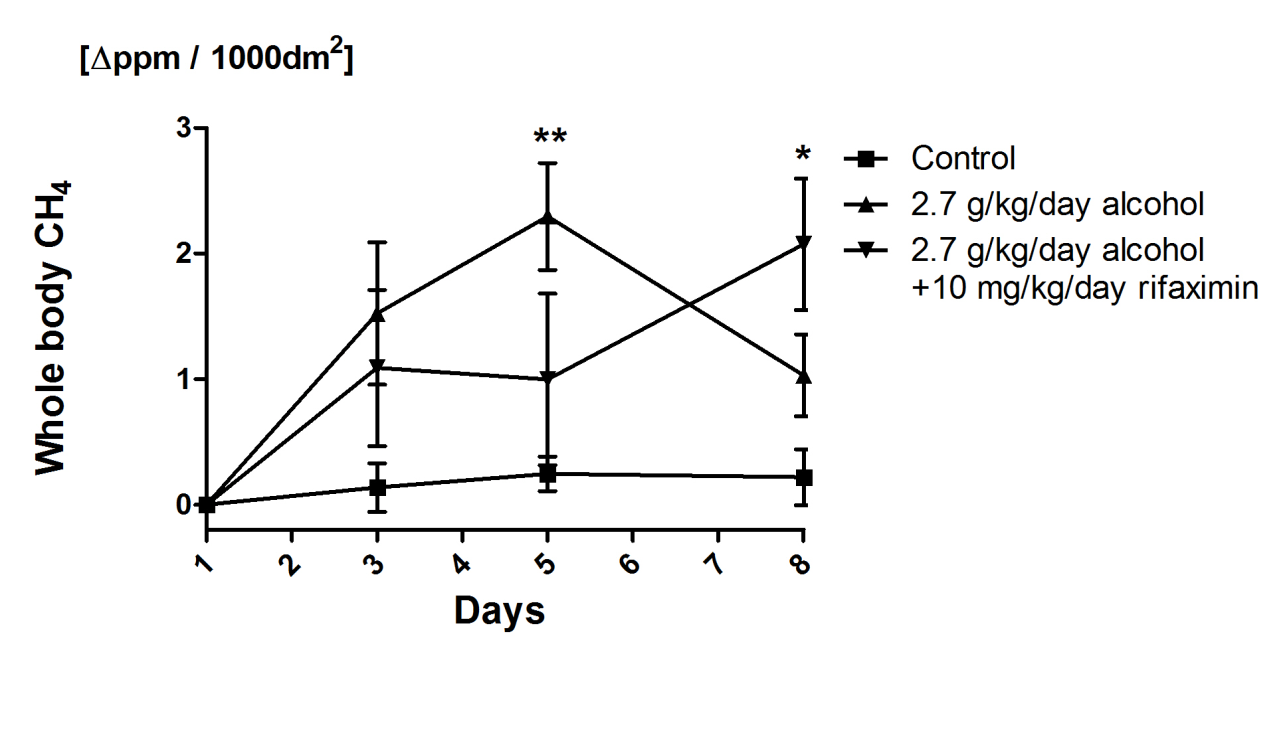


**Supplementary Fig 1.** Whole-body CH_4_ release in ethanol-fed rats with or without antibiotic treatment. CH_4_ production was measured on days 1, 3, 5, and 8 of the investigation. On day 1, all the rats were untreated. Black squares represent the data for the untreated control group. Ethanol feeding induced significant CH_4_ production by the fifth day in the 2.7 g/kg/day alcohol-treated group (black triangle; p<0.01). Upside-down triangles demonstrate the data for 2.7 g/kg/day alcohol + antibiotic-treated group. A statistically significant (p<0.05) increase in CH_4_ release was observed on day 8 in the antibiotic-treated animals that were subjected to the ethanol challenge (day 8 M: 2.153 dppm/1000 dm^2^; p25: 1.666 dppm/1000 dm^2^; p75: 3.503 dppm/1000 dm^2^). Mean values and standard error of mean (SEM) are given; *p<0.05 and **p<0.01 *vs*. control group, Statistics: two-way ANOVA, Bonferroni post-hoc test.

In summary, rifaximin pretreatment initially reduced the increased methane production as compared to the 2.7 g/kg/day ethanol-fed group, but by day 8 the whole-body methane production in the 2.7 g/kg/day ethanol + antibiotics group was significantly higher as compared to untreated controls. In other words, the methane-producing response was present in the antibiotics-treated group, but the course of the reaction was somewhat delayed, a significant increase being observed only at the end of the experimental period. The explanation for this delay might be the experimental design (it is important to note that rearrangements of gut microbiome due to the applied antibiotics treatment cannot be ruled out) or it may be due to the inhibition of the NF-κB-linked ROS-producing pathway of rifaximin via the pregnane X receptor (Refs. Cheng J, *J Pharmacol Exp Ther*, 2010; Mencarelli A, *Eur J Pharmacol,* 2011; Cheng J, *Toxicol Sci*, 2012). This possible anti-oxidant mechanism of action makes the interpretation of the new data difficult, but it reinforces the assumption that ethanol-induced methane production is not solely a by-product of the methanogen aneaerobic flora.

**References**

Finegold, S. M., Molitoris, D. & Väisänen, M. L. Study of the in vitro activities of rifaximin and comparator agents against 536 anaerobic intestinal bacteria from the perspective of potential utility in pathology involving bowel flora. *Antimicrob Agents Chemother* **53**, 281-6 (2009).

Cheng, J. *et al.* Therapeutic role of rifaximin in inflammatory bowel disease: clinical implication of human pregnane X receptor activation. *J Pharmacol Exp Ther* **335**, 32-41 (2010).

Cheng, J., Krausz, K. W., Tanaka, N. & Gonzalez, F. J. Chronic exposure to rifaximin causes hepatic steatosis in pregnane X receptor-humanized mice. *Toxicol Sci* **129**, 456-68 (2012).

Mencarelli, A. *et al.* Inhibition of NF-κB by a PXR-dependent pathway mediates counter-regulatory activities of rifaximin on innate immunity in intestinal epithelial cells. *Eur J Pharmacol* **668**, 317-24 (2011).
